# Spatial genomics and brain spatial transcriptomic atlas for precision medicine

**DOI:** 10.1093/pcmedi/pbag005

**Published:** 2026-02-12

**Authors:** Wei Xiong, Alexander Hindeleh, Charles Wang

**Affiliations:** Department of Otolaryngology, Beijing Friendship Hospital, Beijing 100050, China; Department of Pharmaceutical Sciences, School of Pharmacy and Pharmaceutical Sciences, University of California-Irvine, Irvine, CA 92697, USA; Center for Genomics, School of Medicine, Loma Linda University, Loma Linda, CA 92350, USA

The emergence of spatial genomics has introduced new possibilities for studying structure–function relationships in neuropsychiatric disorders. Spatial transcriptomics allows the detection of thousands of genes simultaneously up to single-cell resolution, providing the spatial location of gene transcriptional activity within a tissue sample. Spatial genomics technologies are developing rapidly, and many of them have been used to construct brain spatial genomics atlases. A brain spatial transcriptomic atlas is a map of the gene expression across brain regions in situ—i.e., within their anatomical context—often at single-cell or near-single-cell resolution. The brain spatial genomics atlas has been constructed for a few species including mice and humans. These brain atlases can be used to further investigate specific genes or pathways that are dysregulated in disease, advancing therapeutic development as a result. Spatial genomics and the brain spatial transcriptomics atlas are vital for driving precision medicine efforts for neurological disorders.

## Spatial genomics and brain spatial transcriptomics atlas for diagnosis and treatment of neurodegenerative diseases, brain neoplasms, and substance use disorders

**Figure 1:**
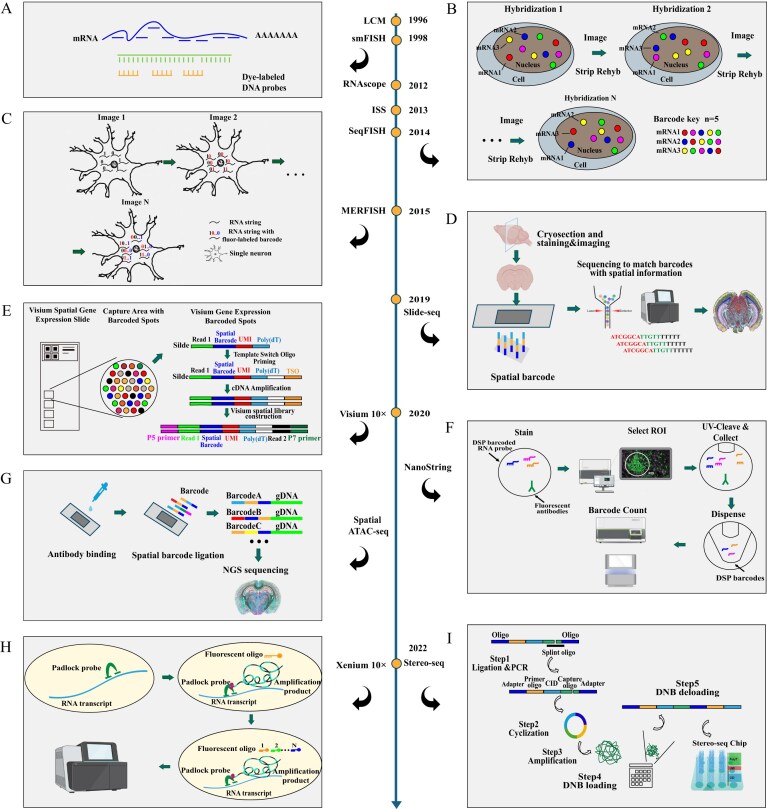
Timeline and schematic diagram of major spatial genomics technologies. The arrow line in the middle shows the name and timeline of the spatial genomics technologies developed. (A) Scheme of smFISH, imaging-based single molecule fluorescence *in situ* hybridization (smFISH). Fluorescently tagged oligo probes label RNA transcripts to study gene expression in subcellular and single-cell resolution. (B) Scheme of sequential fluorescence in situ hybridization (seqFisH), an imaging-based technique combining imaging and molecular barcoding at subcellular and single-cell resolution. (C) Scheme of multiplexed error-robust fluorescence in situ hybridization (MERFISH), one of the highest-throughput imaging-based techniques which implements a novel combinatorial barcoding scheme where each targeted gene in the custom-designed panel is assigned a unique binary barcode, allowing for simultaneous imaging of thousands of genes at subcellular and single-cell resolution. (D) Scheme of Slide-seq, a high-resolution, sequencing-based method for transferring RNA molecules from tissue sections onto a surface with DNA-barcoded beads and known positions, allowing the locations of the RNA to be inferred by sequencing. (E) Scheme of 10x Visium spatial gene expression, which was one of the first sequencing-based spatial transcriptomics methods. It starts from tissue preparation and permeabilization, molecular capture, reverse transcription, library construction, to sequencing and data analysis. Newer versions, such as Visium HD, provide single-cell resolution. (F) Scheme of NanoString CosMx spatial transcriptomics including the utilization of DSP barcoded RNA probes, i.e., probes with fluorescent tags hybridized directly to the target molecules, then the barcodes are read and quantified, providing a direct count of the target molecules with single-cell and subcellular resolution. (G) Scheme of Spatial ATAC-seq starting with tissue sections that are treated with Tn5 transposase, then the tagged DNA fragments are extracted from the tissue sections and amplified to construct a library for sequencing with near single-cell resolution. (H) Scheme of 10x Xenium spatial transcriptomics starting with sections that are mounted to a Xenium slide and then treated with circularizable DNA probes to access the RNA for labeling. After undergoing successive rounds of fluorescent probe hybridizations, an optical signature specific to each gene is generated, enabling target gene identification at single-cell and subcellular resolution. Finally, a spatial map of the transcripts is built across the entire tissue section. (I) Scheme of STOmics Stereo-seq consisting of the following steps: tissue sections are prepared and placed onto a specialized Stereo-seq slide. The slides are designed with an array of DNA nanoballs (DNBs) that act as barcoded capture spots for RNA molecules that are hybridized to the barcoded DNBs on the slide. The captured RNAs are reverse transcribed into cDNA on the slide. The cDNA molecules, tagged with spatial barcodes, are then amplified to create a sequencing library, allowing for near single-cell resolution gene detection.

Spatial genomics has experienced rapid development (Fig. 1) and it allows the detection of a large number of genes simultaneously with spatial context which has led to the construction of the brain spatial genomics atlas [1, 2]. Neurodegenerative diseases such as Alzheimer’s disease (AD) and Parkinson's disease often involve the loss or dysfunction of cells in specific brain regions [3, 4]. Brain atlases constructed using spatial omics can offer spatial context for transcriptomic gene expression with single-cell resolution and epigenomic regulation, neural connectivity, and brain structures, which are crucial for diagnosing and treating neurodegenerative diseases. For example, a spatial brain atlas integrated with gene expression data can show how genes related to neurodegenerative diseases are expressed in different brain regions. In AD, spatial transcriptomics (ST) has revealed critical insights into the gene signatures and pathways associated with Aβ plaques and tau tangles, which are hallmark features of AD [5]. By using the Visium spatial transcriptomics platform, researchers identified spatially distinct neuron–glia interactions in the human middle temporal gyrus, providing insights into disease vulnerability mechanisms and potential biomarker discoveries [6]. They identified layer-specific differentially expressed genes and multiple altered gene co-expression networks, indicating that AD leads to disrupted cell–cell communication within the middle temporal gyrus, including transcriptional alterations near Aβ plaques [6]. This spatial resolution is not offered by dissociative single-cell transcriptomics, positioning ST as a valuable method for understanding region-specific cellular dynamics in disease states. Since AD patients experience different symptoms, insights into differentially expressed genes within layers, regions, and specific cell-types may eventually guide patient stratification for clinical trials, strengthening precision medicine efforts for AD.

ST is also highly effective for identifying biomarkers specific to different stages of neurodegeneration. This is especially important for diseases like AD, where early intervention may slow down cognitive decline. For example, Chen et al. investigated the transcriptional changes in tissue domains around amyloid plaques using ST in an AD mouse model. Compared with the normal mouse brain, early alterations were observed in a gene co-expression network enriched with myelin and oligodendrocyte genes, and in the later phase of the disease, a multicellular gene co-expression network involving 57 plaque-induced genes was found [7]. As brain ST atlases are developed at various disease stages, researchers can identify stage-specific biomarkers to guide more personalized treatment plans.

Recent advances in spatial genomics have greatly impacted the diagnosis and treatment of brain neoplasms, particularly in understanding complex tumors like glioblastoma. Spatial genomics enables researchers to map the molecular and cellular landscapes of brain tumors, revealing how cancer cells interact with their microenvironment. This detailed view helps identify new therapeutic targets by understanding the heterogeneity and cellular states within tumors. For instance, the GBM-space project focuses on glioblastoma multiforme (GBM), one of the most aggressive brain cancers. Using ST and single-cell RNA-sequencing, researchers have uncovered the diverse cellular states within these tumors, which can shift in response to treatment and contribute to therapy resistance [8]. This spatial information can guide the development of more precise therapies aimed at specific tumor subregions or cell types.

Studies have shown that ST helps map interactions between cancer cells and the tumor microenvironment. Identifying how immune cells, like tumor-associated macrophages, influence the tumor’s growth or suppression helps in designing better immunotherapies [8]. Gliomas exhibit spatial heterogeneity, meaning that different parts of the tumor have varying genetic mutations or expression profiles. Spatial genomics and the brain ST atlas enable researchers and clinicians to map the tumor in relation to the surrounding normal brain tissue, to guide targeted treatments [9]. Besides that, during glioma surgery, preserving brain function while removing as much tumor tissue as possible is essential. A brain ST atlas that includes functional mapping helps neurosurgeons avoid critical areas related to speech, motor function, and cognition, which is crucial in minimizing postoperative complications and improving patient outcomes. An advanced brain ST atlas serves as a platform for clinical research by providing standardized reference data.

The National Survey on Drug Health and Use reported that >40 million people in the USA had been diagnosed with substance use disorder. Spatial genomics and the brain ST atlas are powerful tools for delineating the spatial locations of key reward-related genes in the brain, and for streamlining further research investigations on the mechanisms underlying drug addiction.

A recent study by Smith et al. outlined the use of ST in identifying a rare population of glutamatergic neurons in the dorsal peduncular nucleus that has implications for mediating reward-aversion states in opioid use in mice [10]. Opioids are either natural or synthetic pain relievers with a high addiction potential. In the Smith et al. study, they utilized the Allen Mouse Brain Expression Atlas to validate their spatial-sequencing findings and were able to confirm the presence of the rare neuron population, which can guide future studies of the dorsal peduncular nucleus for relevant genes or pathways [10]. This study demonstrates the importance of reference brain ST atlases in guiding disease-relevant cell-type identification and biomarker discovery to advance precision medicine efforts for opioid use disorder.

## Prospects

Spatial genomics is a transformative technology that provides a 4D view of biology, integrating space, time, gene expression, and cellular interactions. It has strong potential to reshape biomedical research, disease diagnosis and therapy, drug discovery, and precision medicine. Further progress is needed before brain ST atlases can be applied clinically, including reducing costs, establishing uniform technical standards, achieving subcellular and single-cell resolution across platforms, and standardizing data analysis pipelines. As ST technologies and analysis algorithms improve, and the field recognizes the impact of these atlases, the prospects of standardized, high-throughput brain ST atlases will become a reality, bringing positive outcomes for the field of precision medicine.

Beyond their roles in brain diseases, brain cancers, and substance use disorders, the brain spatial genomics atlas offers broader prospects. First, it will deepen our understanding of brain development and function by mapping cell-type emergence and specialization, revealing spatial gene expression patterns underlying cognition and behavior, and improving models of neural circuits and regional specialization. Second, it will help reveal the underlying mechanisms of brain disease and the discovery of new biomarkers, e.g., by allowing spatial localization of gene expression changes in disorders like AD, Parkinson's disease, schizophrenia, autism, and brain cancers; identifying disease-specific cell populations and microenvironments; and enabling the discovery of spatial biomarkers for early detection and targeted therapy. Third, it will be a great tool and resource for comparative neuroscience and evolution studies, e.g., facilitating comparison between species (human versus mouse or non-human primates) to shed light on the evolution of brain complexity, helping identify conserved and unique gene expression patterns across species. Finally, it will support precision medicine by guiding region- and cell-specific drug delivery, gene therapy, and personalized interventions based on individual spatial gene expression profiles.
